# Telemedicine for Patients With Systemic Lupus Erythematosus in a Publicly Funded Hospital System: Retrospective Study

**DOI:** 10.2196/49065

**Published:** 2024-11-01

**Authors:** Sebastian Bruera, Kristen Andrews Staggers, Maria Eugenia Suarez-Almazor, Sandeep Krishna Agarwal

**Affiliations:** 1 Department of Immunology, Allergy, and Rheumatology Baylor College of Medicine Houston, TX United States; 2 Department of Health Services Research MD Anderson Cancer Center University of Texas Houston, TX United States

**Keywords:** lupus, systemic lupus erythematosus, telemedicine, COVID-19, access to care, autoimmune disease, no-show, socioeconomic status, adherence, laboratory test, management

## Abstract

**Background:**

Systemic lupus erythematosus (SLE) is a chronic autoimmune disease that requires frequent clinic and laboratory visits. However, patients with SLE, particularly those who are underresourced, have unacceptably high rates of no-shows.

**Objective:**

This study aims to determine no-show rates associated with telemedicine visits during the COVID-19 pandemic in comparison to no-show rates associated with contemporaneous and historic in-person visits.

**Methods:**

We performed a retrospective cohort study in a publicly funded county hospital system in Houston, Texas. We identified a cohort of established patients with SLE by the *International Classification of Diseases* codes that were independently confirmed as SLE by a review of medical records. We identified patients who were seen from March to December in 2018, 2019, and 2020 (to reflect the height of the COVID-19 pandemic and account for seasonal changes in disease activity). Our primary outcome was the percentage of no-shows for rheumatology clinic appointments. Our secondary outcome was laboratory use adherence, which was defined as lupus-specific blood and urine studies conducted within 30 days of the scheduled appointment. Covariates included age, sex, race, ethnicity, and SLE-related prescription drugs.

**Results:**

We included 156 patients with SLE in our analysis. Most were female (n=141, 90.4%), were Hispanic (n=75, 49.3%), and had a median age of 43 (range 19-80) years. In 2020, the no-show rate for telemedicine was 5.5% (10/182) compared to a no-show rate of 16.2% (31/191) for in-person visits (*P*=.002). After multivariable adjustment for covariates, the odds of no-show were lower for telemedicine visits (odds ratio 0.39, 95% CI 0.20-0.77). There were no differences in adherence to laboratory testing.

**Conclusions:**

Telemedicine visits had decreased odds of no-shows without difference in laboratory testing adherence after adjustment for covariates. More research is needed to determine the clinical impact of telemedicine on patients with SLE.

## Introduction

The COVID-19 pandemic triggered widespread and emergent use of telemedicine as an option for patients to avoid exposure to the SARS-CoV-2 virus [1,2]. The use of telemedicine has been especially important in patients with chronic diseases, such as systemic lupus erythematosus (SLE), who are at a high risk of severe COVID-19 and may benefit from less public exposure [3,4]. Missed appointments in general are associated with an increased risk of mortality and adverse outcomes [5-7]. Telemedicine has the potential benefit of improving no-shows by making clinic visits more accessible. This may be particularly important for patients of lower socioeconomic status (SES) who may have difficulties attending visits because of transportation, work, or financial factors. Yet, it has been suggested that telemedicine services are less likely to be used in populations with lower SES [8,9].

Despite the potential advantages of telemedicine, especially for patients of low SES, there are still important concerns, which should be considered when telemedicine is implemented for patients with SLE. First, patients with SLE are usually followed every 3 to 4 months and require serial evaluations including blood pressure monitoring, physical examination, and clinical laboratory tests—regardless of the presence or absence of symptoms or examination findings [10]. It is unclear how telemedicine may affect adherence to visits and adherence to laboratory testing since patients may be more likely to obtain blood work if they are already at the clinic.

We performed a retrospective cohort study among patients with SLE managed in a county hospital system in Houston, Texas. This population is highly diverse, with most patients being underinsured or uninsured and having low SES. We hypothesized that patients with SLE in this system will have lower no-show rates with telemedicine modalities, such as telephone and video visits, compared to in-person visits. Furthermore, we explored patient characteristics (such as medications and age) and their association with visit types. We also examined the impact of telemedicine on patients’ adherence to laboratory testing.

## Methods

### Design and Patient Population

We performed a retrospective analysis of patients with SLE seen in the Harris Health System (HHS). The HHS is a fully integrated health care system that provides care to residents of Harris County, Texas, which has an estimated population of 4.7 million [11]. The patient population at the HHS includes 54% who are uninsured, 22% who have Medicaid, 12% who have Medicare, and 13% who have private insurance. Most uninsured patients qualify for HHS insurance (“the gold card”) that provides partial or full reimbursement for care to patients with a household income that does not exceed 150% of the federal poverty level. Once a patient receives a gold card, the copay for any visits is dictated by income stratification and ranges.

### Patient Selection

We identified a cohort of established patients with SLE using the *International Classification of Diseases, 10th Revision* diagnostic codes (M32.x, excluding M32.0). Patients with SLE were included if they were seen at the HHS rheumatology clinics by a rheumatologist at least once between March 2020 and September 2020 (at a time when telemedicine was implemented because of the pandemic). Data were initially collected through the information technology services provided by the HHS. Patients were seen at a large HHS rheumatology teaching clinic staffed by 7 rheumatologists. The diagnosis of SLE was independently confirmed by chart review by a rheumatologist (SB) if they met the American College of Rheumatology 2019 diagnostic criteria. Patients were offered telemedicine visits either by telephone encounters or by video with a secured third-party platform (Doximity). Between March 2020 and September 2020, the HHS rheumatology clinic offered both in-person visits along with telemedicine encounters. The decision to have a telemedicine versus in-person encounter was driven by patient preference.

As controls, we identified cohorts of patients with SLE seen in the HHS rheumatology clinic from March to September of 2018 and 2019 (before the pandemic). We limited the control cohort to patients with SLE seen from March to September to account for potential seasonal changes in practice patterns and disease activity, which have been previously described [12]. We also identified a subgroup for analysis of patients who were seen at least once in both 2019 and 2020. This subgroup analysis of “no-shows” was performed to examine trends for the same patients who had attended at least 1 follow-up appointment each year.

### Outcomes

Our primary outcome was the percentage of no-shows for rheumatology clinic follow-up appointments. No-shows were defined as visits for which patients did not show up or that were canceled by the patient within the same day. Clinic visits rescheduled by patients prior to 24 hours before the clinic appointment were not considered no-shows. Secondary outcomes included laboratory testing such as complete blood count, comprehensive metabolic panel, urinalysis, serum complement levels (C3 and C4), and serum titers of anti–double-stranded DNA (anti-dsDNA) antibodies within 30 days before or after each completed clinic visit. It is the standard in rheumatology clinics that all patients with SLE obtain laboratory testing and have a clinic appointment at least every 3 months, regardless of disease activity [10]. All laboratory studies for patients seen at HHS clinics are done within the HHS at 1 of 17 clinics or 2 large hospitals. Laboratory tests can be ordered as a preclinic laboratory test (performed within 14 days of a clinic appointment) or obtained on the day of their clinic visit. For telemedicine encounters, blood work can be obtained before the clinic or at patients’ convenience for any day of their preference at the closest HHS clinical laboratory.

### Covariates

Baseline demographics included age, sex, race, ethnicity, and use of SLE-related prescribed drugs at the initial visit in the period of interest. We included baseline demographics as a covariate due to multiple studies showing differences in digital literacy among patients of different ages, races, and ethnicities [13]. The insurance coverage for each specific visit appointment was not available, as insurance status can change over time; however, as previously mentioned, over 85% of patients that are seen in our clinics are uninsured or publicly insured.

We also included whether patients were prescribed SLE-specific drugs (hydroxychloroquine, mycophenolate, azathioprine, methotrexate, rituximab, belimumab, tacrolimus, prednisone, and cyclophosphamide) in 2018, 2019, or 2020. Our data included medications prescribed by providers at each visit but did not include whether patients had filled prescriptions (ie, could not measure adherence). Some patients may have a 6-month active prescription for a drug that may not necessarily be refilled at a 3-month follow-up visit. Due to this, we included SLE-specific drug prescriptions as a variable of whether the patient was ever (at any 1 time point) prescribed (or refilled) a medication throughout the year (2018, 2019, or 2020) as opposed to by visit. We also included codes for infusions of rituximab, belimumab, and cyclophosphamide. We assumed that drug therapies may be an important covariate as some drugs, for instance, mycophenolate mofetil, require more frequent laboratory monitoring than others, such as hydroxychloroquine. We did not use drug therapy as a surrogate for disease activity.

We also used our covariates to determine any associations with visit types in 2020 when telemedicine was more readily available. We divided patients into either all in-person visits, 2 or more telemedicine visits, or in-person visit with 1 telemedicine visit to determine if there were differences between the covariates.

### Statistical Analysis

Patient and visit characteristics were summarized by means with SDs, median with ranges, or frequencies with percentages. Summary statistics were compared between groups using ANOVA, independent 1-tailed *t* tests, median regression, Wilcoxon rank sum, Fisher exact, or chi-square tests according to the type and distribution of each variable of interest. Pairwise tests with Holm *P* value adjustment were done when necessary. Since patients had multiple visits, some comparisons were performed using mixed effects linear regression or generalized estimating equations (GEEs) to account for the correlated data structure as appropriate. GEE models used an exchangeable correlation structure, when possible; otherwise, an independent correlation structure was used. A multiple GEE model was used to determine whether not showing up was associated with visit-type appointments. We included covariates that had statistical significance associated with a no-show or visit type (telemedicine vs in-person). For example, older age and certain medications (methotrexate and belimumab) were more associated with telemedicine visits and introduced into the GEE model. Among patients seen both in 2019 and 2020, GEE was used to determine factors associated with laboratory test adherence (defined as being performed within 1 month of the visit). For laboratory test visits, the first visit was used if a patient had multiple visits less than 6 weeks apart. The GEE models estimated odds ratios (ORs) with 95% CI. We performed the analysis of “no-shows” on our subgroup of patients that were seen in both 2019 and 2020 to account for the same patients that historically followed.

### Ethical Considerations

This retrospective study was approved by the Baylor College of Medicine Institutional Review Board with waived informed consent under protocol number H-45296. As this was a retrospective review, a waiver of consent was granted. Our data were deidentified and all results were stored in secure and encrypted servers at the Baylor College of Medicine. The data collected were deidentified prior to analysis.

## Results

### Baseline Characteristics

Baseline characteristics of included patients are shown in Table 1.

There were 156 patients included in our analysis. Most patients were female (90.4%), were Hispanic (49.3%), had a median age of 43 (range 19-80) years, and had received hydroxychloroquine (n=144, 92%) or prednisone (n=120, 77%) throughout the follow-up period. Baseline characteristics broken down by visit type are shown in Table S1 in Multimedia Appendix 1. We included 771 in-person visits and 182 telemedicine (including telephone or video) visits in our analysis. We found that telemedicine visits were associated with older age (median 45.3, range 19.9-81.5 vs 41.2, range 19.2-82.1 years; *P*=.01) and were less likely to occur in patients who were prescribed mycophenolate (74/182, 40.7% vs 367/771, 47.6%; *P*=.03) or prednisone (142/182, 78% vs 644/771, 83.5%; *P*=.02) compared to in-person visits. Differences in all other characteristics were not statistically significant (all *P*>.05; Table S2 in Multimedia Appendix 1).

**Table 1:** Baseline characteristics of patients with SLE (N=156).

Baseline characteristics			Values
**Sex, n (%)**			
	Female	141 (90.4)	
	Male	15 (9.6)	
**Race (n=150), n (%)**			
	Hispanic	74 (49.3)	
	Non-Hispanic Black	35 (23.3)	
	Non-Hispanic White	17 (11.3)	
	Other	24 (16)	
Age (years), median (range)			43.2 (19.2-79.5)
**Prescription drug use (ever prescribed as per medical record), n (%)**			
	Hydroxychloroquine	144 (92.3)	
	Mycophenolate	63 (40.4)	
	Azathioprine	41 (26.3)	
	Methotrexate	23 (14.7)	
	Rituximab	4 (2.6)	
	Belimumab	16 (10.3)	
	Tacrolimus	8 (5.1)	
	Prednisone	120 (77)	
	Cyclophosphamide	5 (3.2)	

### Patient Characteristics Associated With Visit Type

We determined differences in patient characteristics between those who had all in-person visits, 2 or more telemedicine visits, or an in-person visit and 1 telemedicine visit in 2020 (Table 2).

Patients who had 2 or more telemedicine appointments were less likely to be prescribed methotrexate (3/63, 4.8% vs 11/54, 20.4%; Holm adjusted *P* value=.03) or prednisone (27/63, 42.9% vs 37/54, 68.5%; Holm adjusted *P* value=.03) during the year compared to those with in-person appointments or only 1 telemedicine appointment. Whether the patient was prescribed mycophenolate was significantly different between visit types (*P*=.05), but when performing pairwise comparisons on each visit type category, none of them were significant (all *P*>.05). No patients had received cyclophosphamide in this study.

**Table 2:** Patient characteristics associated with visit types in 2020.

Patient characteristics		All in-person visits (n=21)	2 or more telemedicine visits^a^ (n=63)	In-person visit + 1 telemedicine visit (n=54)	*P* value^b^		*P* value , significant pairwise comparison results^c^	
**Age (years; first 2020 visit), median (IQR)**		36.7 (33.1-53)	45.1 (20.6-77.0)	45.2 (19.8-81.5)	.25		N/A^d^	
**Sex, n (%)**								
	Female	19 (90.5)	59 (93.7)	49 (90.7)	.76		N/A	
**Race, n (%)^e^**						.08		
	Hispanic	6 (30)	32 (52.5)	25 (49)			N/A	
	Non-Hispanic Black	9 (45)	13 (21.3)	11 (21.6)			N/A	
	Non-Hispanic White	4 (20)	3 (4.9)	5 (9.8)			N/A	
	Others	1 (5)	13 (21.3)	10 (19.6)			N/A	
**Medications, n (%)**								
	Hydroxychloroquine	N/A	47 (74.6)	44 (81.5)	.67		N/A	
	Mycophenolate	9 (42.9)	15 (23.8)	24 (44.4)	.05		NS^f^	
	Azathioprine	5 (23.8)	11 (17.5)	14 (25.9)	.52		N/A	
	Methotrexate	3 (14.3)	3 (4.8)	11 (20.4)	.02		.03^g^	
	Rituximab	0 (0)	1 (1.6)	1 (1.9)	>.99		N/A	
	Belimumab	1 (4.8)	3 (4.8)	2 (3.7)	>.99		N/A	
	Tacrolimus	0 (0.0)	3 (4.8)	2 (3.7)	.85		N/A	
	Prednisone	15 (71.4)	27 (42.9)	37 (68.5)	.008		.03^g^	

^a^This includes patients who have only 1 visit in 2020, and that visit is a telemedicine visit.

^b^Calculated using median regression test, Fisher exact test, or chi-square test.

^c^Pairwise Fisher exact test with Holm *P* value adjustment.

^d^N/A: not applicable.

^e^All in-person visits: n=20; two or more telemedicine visits: n=61; and in-person visit + 1 telemedicine visit: n=51.

^f^NS: Not significant.

^g^Two or more telemedicine visits versus in-person visit + 1 telemedicine visit.

### No-Shows for Clinic Visits

All clinic visits from March to September in 2018 and 2019 were in-person (275 in 2018 and 305 in 2019). From March 2020 to September 2020, of out a total of 373 visits, there were 191 (51.2%) in-person visits and 182 (48.8%) telemedicine visits (Table 3). There was no statistical difference in the no-show rates between in-person visits in 2018, 2019, and 2020 (31/275, 11.3% vs 38/305, 12.5% vs 31/191, 16.2%, respectively). In 2020, when telemedicine was implemented, the no-show rate for in-person visits was 16.2% (31/191) versus 5.5% (10/182) for telemedicine visits (*P*=.002). We used independent GEEs to determine any characteristics associated with no-shows (Table S3 in Multimedia Appendix 1). After adjusting for age and significant SLE prescription drugs (methotrexate and belimumab) in a multiple GEE, there was a significantly decreased odds of no-shows for telemedicine versus in-person clinic appointments (adjusted OR 0.39, 95% CI 0.20-0.77; *P*=.007; Table S4 in Multimedia Appendix 1).

We also performed a subgroup analysis on patients who were seen at least once in 2019 and 2020. There were 300 visits in 2019 and 332 visits in 2020. The total no-show rates between 2019 and 2020 were similar (38/300, 11% vs 41/332, 10.5%; *P*=.85). Among these visits, we also found that telemedicine appointments had significantly lower odds of no-show compared to in-person appointments (adjusted OR 0.31, 95% CI 0.14-0.69) when adjusting for those SLE prescription drugs that were significantly different (only rituximab) according to the type of visit.

**Table 3:** Visit characteristics stratified by type of visit.

Visit characteristics		In-person visits (n=771)	Telephone visits (n=157)	Video visits (n=25)	Telephone or video visits (n=182)	Total, n (%)
**Year, n (%)**						
	2018 (n=275)	275 (100)	0 (0)	0 (0)	0 (0)	275 (100)
	2019 (n=305)	305 (100)	0 (0)	0 (0)	0 (0)	305 (100)
	2020 (n=373)	191 (51.2)	157 (42.1)	25 (6.7)	182 (48.8)	373 (100)
**No-shows, n/N (%)**						
	2018	31/275 (11.3)	N/A^a^	N/A	N/A	31/275 (11.3)
	2019	38/305 (12.5)	N/A	N/A	N/A	38/305 (12.5)
	2020	31/191 (16.2)	7/157 (4.5)	3/25 (12)	10/182 (5.5)	41/373 (11)

^a^N/A: not applicable.

### Laboratory Test Use

When comparing laboratory test use between 2019 and 2020, the only significant difference was in urinalysis which was more frequently performed for telemedicine visits than in-person visits (38/289, 13.1% vs 7/257, 2.7%; *P*<.001; Table 4). We also compared the use of laboratory tests between in-person and telemedicine visits in 2020 using GEE. No statistically significant differences were observed (all *P*>.05). We found that there were no differences in nonadherence to laboratory testing for all laboratory tests, although there was a trend toward significance for anti-dsDNA testing (4/136, 2.9% nonadherence for in-person visits vs 13/153, 8.5% nonadherence for telemedicine visits; *P*=.06; Table 4), but it was not statistically significant. We found that urine studies had the highest proportion of nonadherence (16/136, 11.8% for in-person visits vs 22/153, 14.4% for telemedicine visits; *P*=.51), although this could be explained by other factors not measured such as end-stage renal disease.

**Table 4:** Nonadherence to laboratory testing for completed visits in 2019 and 2020.

Laboratory studies not completed within 30 days of appointment	Total visits in 2019 (n=257), n (%)	Total visits in 2020 (n=289), n (%)	*P* value^a^	In-person visits in 2020 (n=136), n (%)	Telemedicine visits in 2020 (n=153), n (%)	*P* value^a^
CBC^b^	7 (2.7)	4 (1.4)	.28	0 (0)	4 (2.6)	N/A^c^
BMP^d^ or CMP^e^	5 (1.9)	7 (2.4)	.71	2 (1.5)	5 (5.3)	.33
Urinalysis	7 (2.7)	38 (13.1)	<.001	16 (11.8)	22 (14.4)	.51
Anti-dsDNA^f^	19 (7.4)	17 (5.9)	.48	4 (2.9)	13 (8.5)	.06
Complements	9 (3.5)	18 (6.2)	.15	5 (3.7)	13 (8.5)	.10

^a^Calculated using a generalized estimating equation with an independent correlation structure.

^b^CBC: complete blood count.

^c^N/A: not applicable.

^d^BMP: basic metabolic panel.

^e^CMP: comprehensive metabolic panel.

^f^Anti-dsDNA: anti–double-stranded DNA.

## Discussion

We evaluated adherence to telemedicine visits in the management of patients with SLE, at a publicly funded county hospital serving primarily underserved patients. We also determined whether there were differences in laboratory use between patients who received telemedicine versus in-person visits. Our results demonstrate that telemedicine encounters had significantly lower odds of no-shows compared to in-person encounters. We also found that no-show rates were similar for 2019 and 2020 despite the emergence of the COVID-19 pandemic, which could be due to the availability of telemedicine, as no-shows for telemedicine versus in-person in 2020 were significantly lower (10/182, 5.5% vs 31/191, 16.2%). Furthermore, to our knowledge, this is the first study that shows telemedicine visits do not affect laboratory use within 30 days of the clinic visits.

Studies have shown that telemedicine can play a role in the management of chronic diseases that require frequent clinic visits [14]. Other studies in SLE have shown that telemedicine was used as frequently as in-person visits during the initial COVID-19 pandemic, although this is the first study to demonstrate that this occurred in an underresourced patient population [15,16]. Of note, the widespread use of telemedicine is also seen in patients with severe chronic diseases such as SLE. A recently published randomized controlled trial in Hong Kong found that the use of telemedicine in patients with lupus nephritis was associated with more hospitalizations [17]. Our study did not address disease activity or hospitalization, and further research is needed to assess how the widespread use of telemedicine may impact these factors.

Our study is consistent with studies in other populations that suggest that telemedicine may provide advantages for underserved populations by decreasing missed appointments. One systematic review of 28 studies reported on the use of telehealth for patients from racial and ethnic minority populations. Results showed that the implementation of telehealth improved access to care; however, there were still barriers related to the technology needed for telemedicine [13]. A separate study using administrative claims data also examined the use of telemedicine in general patient populations and found that telemedicine was associated with fewer missed appointments [18]. However, this study did not include patient populations such as SLE that require frequent clinic visits and laboratory studies (at least every 3 to 4 months). Although our study suggests that telemedicine may be a strategy to decrease the no-show rate in patients with low SES and SLE, more research is needed to determine how other characteristics (including primary language, digital literacy, and disease activity) influence telemedicine and potentially disease outcomes. Furthermore, telemedicine should now be studied as the COVID-19 pandemic has entered the endemic phase.

The strength of our study includes a large number of patients with SLE of low SES in 1 large hospital system where all clinic appointments and laboratory values are documented. There are several limitations in our study. First, we had a predominance of telephone encounters compared to video visits, albeit this has also been seen in other studies, especially among patients of Black and Hispanic ethnicity and of low SES, which were the majority [19]. The use of video visits may affect the no-show rate by presenting technological challenges. Second, our study did not adjust for disease severity according to validated indices as it was retrospective, and it only used prescription drug use as a surrogate for severity. Finally, we were unable to adequately control for insurance type at the time of the scheduled appointment as this information is not updated regularly; however, we do not expect this to change the results as over 85% of patients in the HHS are publicly insured or uninsured.

In conclusion, our study shows that the use of telemedicine during the initial phase of the COVID-19 pandemic was associated with a low rate of no-shows in a population of underserved patients with SLE without impacting laboratory use. To our knowledge, this is the first study to demonstrate that in patients with SLE telemedicine is not associated with decreased laboratory screening, which is a critical component to the care of patients with lupus. As such, we believe the results of this study warrant further investigation to determine the clinical impact of telemedicine on SLE in prospective studies, as the design of this study was not able to capture important clinical characteristics that may influence telemedicine and clinical outcomes, including digital literacy and disease activity.

## Appendix

Multimedia Appendix 1 Baseline characteristics by visit type, *P* value testing results, and multiple generalized estimating equation odds ratios for no-shows.

## Abbreviations

Anti-dsDNA: anti–double-stranded DNA
GEE: generalized estimating equation
HHS: Harris Health System
OR: odds ratio
SES: socioeconomic status
SLE: systemic lupus erythematosus

## Data Availability

The datasets generated during this study are not publicly available because the participants of this study did not give written consent for their data to be shared publicly but are available from the corresponding author on reasonable request.
