# Supplementary material for: Telemedicine for Patients With Systemic Lupus Erythematosus in a Publicly Funded Hospital System: Retrospective Study
Source: Interact J Med Res. 2024 Nov 1;13:e49065. doi: 10.2196/49065 (PMC11568399; doi:10.2196/49065)
Supplement: Multimedia Appendix 1 [file ijmr_v13i1e49065_app1.docx]

**Table S1.** Visit characteristics stratified by visit type.

| \|  \| In-person \| Video or telephone \|  \| \| --- \| --- \| --- \| --- \| \| Variable \| (N =771) \| (N =182) \|  \| |
| --- | --- | --- | --- | --- | --- | --- | --- | --- |
| \|  \|  \| N \| Median \| (Min, Max) \| N \| Median \| (Min, Max) \|  \| \| --- \| --- \| --- \| --- \| --- \| --- \| --- \| --- \| --- \| \| Age at visit (years) \| \| 771 \| 41.2 \| [19.2,82.1] \| 182 \| 45.3 \| [19.9,81.5] \|  \| \|  \|  \|  \|  \|  \|  \|  \|  \|  \| |
| \|  \|  \| N total \| N \| (%) \| N total \| N \| (%) \|  \| \| --- \| --- \| --- \| --- \| --- \| --- \| --- \| --- \| --- \| \| Gender \| \|  \|  \|  \|  \|  \|  \|  \| \| Female \| \| 771 \| 694 \| (90.0) \| 182 \| 169 \| (92.9) \|  \| \| Male \| \| 771 \| 77 \| (10.0) \| 182 \| 13 \| (7.1) \|  \| \|  \|  \|  \|  \|  \|  \|  \|  \|  \| \| Race \| \|  \|  \|  \|  \|  \|  \|  \| \| White, non-Hispanic \| \| 730 \| 84 \| (11.5) \| 172 \| 12 \| (7.0) \|  \| \| Hispanic \| \| 730 \| 366 \| (50.1) \| 172 \| 95 \| (55.2) \|  \| \| Black, non-Hispanic \| \| 730 \| 169 \| (23.2) \| 172 \| 33 \| (19.2) \|  \| \| Multiracial or Other \| \| 730 \| 111 \| (15.2) \| 172 \| 32 \| (18.6) \|  \| \|  \|  \|  \|  \|  \|  \|  \|  \|  \| \| Ever prescribed Hydroxychloroquine \| \|  \|  \|  \|  \|  \|  \|  \| \| No \| \| 771 \| 68 \| (8.8) \| 182 \| 9 \| (4.9) \|  \| \| Yes \| \| 771 \| 703 \| (91.2) \| 182 \| 173 \| (95.1) \|  \| \|  \|  \|  \|  \|  \|  \|  \|  \|  \| \| Ever prescribed Mycophenolate \| \|  \|  \|  \|  \|  \|  \|  \| \| No \| \| 771 \| 404 \| (52.4) \| 182 \| 108 \| (59.3) \|  \| \| Yes \| \| 771 \| 367 \| (47.6) \| 182 \| 74 \| (40.7) \|  \| \|  \|  \|  \|  \|  \|  \|  \|  \|  \| \| Ever prescribed Azathioprine \| \|  \|  \|  \|  \|  \|  \|  \| \| No \| \| 771 \| 537 \| (69.6) \| 182 \| 133 \| (73.1) \|  \| \| Yes \| \| 771 \| 234 \| (30.4) \| 182 \| 49 \| (26.9) \|  \| \|  \|  \|  \|  \|  \|  \|  \|  \|  \| \| Ever prescribed Methotrexate \| \|  \|  \|  \|  \|  \|  \|  \| \| No \| \| 771 \| 648 \| (84.0) \| 182 \| 158 \| (86.8) \|  \| \| Yes \| \| 771 \| 123 \| (16.0) \| 182 \| 24 \| (13.2) \|  \| \|  \|  \|  \|  \|  \|  \|  \|  \|  \| \| Ever prescribed Rituximab \| \|  \|  \|  \|  \|  \|  \|  \| \| No \| \| 771 \| 750 \| (97.3) \| 182 \| 174 \| (95.6) \|  \| \| Yes \| \| 771 \| 21 \| (2.7) \| 182 \| 8 \| (4.4) \|  \| \|  \|  \|  \|  \|  \|  \|  \|  \|  \| \| Ever prescribed Belimumab \| \|  \|  \|  \|  \|  \|  \|  \| \| No \| \| 771 \| 672 \| (87.2) \| 182 \| 165 \| (90.7) \|  \| \| Yes \| \| 771 \| 99 \| (12.8) \| 182 \| 17 \| (9.3) \|  \| \|  \|  \|  \|  \|  \|  \|  \|  \|  \| \| Ever prescribed Tacrolimus \| \|  \|  \|  \|  \|  \|  \|  \| \| No \| \| 771 \| 716 \| (92.9) \| 182 \| 171 \| (94.0) \|  \| \| Yes \| \| 771 \| 55 \| (7.1) \| 182 \| 11 \| (6.0) \|  \| \|  \|  \|  \|  \|  \|  \|  \|  \|  \| \| Ever prescribed Prednisone \| \|  \|  \|  \|  \|  \|  \|  \| \| No \| \| 771 \| 127 \| (16.5) \| 182 \| 40 \| (22.0) \|  \| \| Yes \| \| 771 \| 644 \| (83.5) \| 182 \| 142 \| (78.0) \|  \| \|  \|  \|  \|  \|  \|  \|  \|  \|  \| \| Ever prescribed Cyclophosphamide \| \|  \|  \|  \|  \|  \|  \|  \| \| No \| \| 771 \| 734 \| (95.2) \| 182 \| 174 \| (95.6) \|  \| \| Yes \| \| 771 \| 37 \| (4.8) \| 182 \| 8 \| (4.4) \|  \| \|  \|  \|  \|  \|  \|  \|  \|  \|  \| \|  \|  \|  \|  \|  \|  \|  \|  \|  \| |
|  |

**Table S2:** P-values testing whether any patient or visit characteristics are associated with visit type (independent GEEs)

|  | **p-value** |
| --- | --- |
| Age | 0.012 |
| Gender | 0.232 |
| Race | 0.065 |
| Ever prescribed Hydroxychloroquine | 0.059 |
| Ever prescribed Mycophenolate | 0.029 |
| Ever prescribed Azathioprine | 0.174 |
| Ever prescribed Methotrexate | 0.331 |
| Ever prescribed Rituximab | 0.117 |
| Ever prescribed Belimumab | 0.121 |
| Ever prescribed Tacrolimus | 0.626 |
| Ever prescribed prednisone | 0.024 |
| Every prescribed Cyclophosphamide | 0.715 |

**Table S3.** P-values testing whether any patient or visit characteristics are associated with no-shows (independent Generalized Estimating Equations).

| **Variable** | **p-value** |
| --- | --- |
| Age at visit (years) | 0.082 |
| Video or telephone visit (vs in-person) | 0.006 |
| Gender | 0.633 |
| Race | 0.103 |
| During COVID-19 (vs before) | 0.615 |
| Ever prescribed Hydroxychloroquine | 0.467 |
| Ever prescribed Mycophenolate | 0.129 |
| Ever prescribed Azathioprine | 0.188 |
| Ever prescribed Methotrexate | 0.041 |
| Ever prescribed Rituximab | 0.671 |
| Ever prescribed Belimumab | 0.043 |
| Ever prescribed Tacrolimus | 0.205 |
| Ever prescribed Prednisone | 0.792 |
| Ever prescribed Cyclophosphamide | 0.009 |

**Table S4.** Multiple Generalized Estimating Equation Odds Ratios for No-Shows.

|  | **Adj. OR** | **95% Confidence Interval** | | **p-value** |
| --- | --- | --- | --- | --- |
| Video or telephone visit (vs in-person) | 0.39 | 0.20 | 0.77 | 0.007 |
| Age at visit | 0.99 | 0.97 | 1.01 | 0.418 |
| Ever prescribed Methotrexate | 0.53 | 0.22 | 1.26 | 0.151 |
| Ever prescribed Mycophenolate | 1.33 | 0.84 | 2.11 | 0.227 |
| Ever prescribed Belimumab | 0.36 | 0.13 | 1.02 | 0.054 |
| Ever prescribed Prednisone | 1.05 | 0.56 | 1.96 | 0.881 |
| Ever prescribed Cyclophosphamide | 2.39 | 1.04 | 5.47 | 0.040 |
